# Electroconvulsive Therapy and (Es)Ketamine in Treatment-Resistant Depression: A Review of Current Evidence

**DOI:** 10.1192/j.eurpsy.2026.11772

**Published:** 2026-07-31

**Authors:** J. Marta, Â. Ferreira, C. Batista, T. Cardoso, A. F. Reis, M. A. Aleixo

**Affiliations:** Arrábida Local Health Unit, Setúbal, Portugal

## Abstract

**Introduction:**

Treatment-Resistant Depression (TRD) is a major clinical challenge, affecting about 30% of patients with major depressive disorder. It is associated with high morbidity and mortality, reflected in increased suicide risk, hospitalizations, and impaired quality of life. Electroconvulsive Therapy (ECT) remains the therapeutic gold standard, particularly in severe cases with psychotic symptoms or high suicidal risk. However, cognitive side effects, stigma, and logistical barriers have encouraged the exploration of alternative and combined treatments, notably Ketamine and Esketamine.

**Objectives:**

This review aims to review the current evidence on the efficacy and safety of Ketamine and Esketamine in combination with ECT for TRD.

**Methods:**

Evidence-based review, conducted through a search on the PubMed database, selecting the most relevant studies published in the last twenty years.

**Results:**

Randomized controlled trials suggest that Ketamine is not inferior to ECT in TRD without psychotic features, showing a better side effect profile and less cognitive impact. A recent meta-analysis indicates ECT remains superior in overall efficacy, although Ketamine/Esketamine provides a faster antidepressant response and may be preferred in specific clinical contexts. The combination of Ketamine or Esketamine with ECT has yielded mixed results. Some evidence suggests potential benefits in extreme refractoriness, though superiority over monotherapy remains unproven. A retrospective study of 30 participants found no significant differences in response or remission rates but suggested potential utility in patients unresponsive to either therapy alone. Intermittent strategies may enhance and prolong antidepressant effects while minimizing cognitive impact. Higher Ketamine doses (>0.8 mg/kg), used as an anesthetic agent during ECT have been linked to faster, stronger antidepressant response and longer seizure, but increased adverse effects and no cognitive advantage. Meta-analyses and systematic reviews indicate that the combination may accelerate the acute antidepressant response, but sustained long-term superiority or relapse prevention has not been demonstrated.

**Conclusions:**

Combining ECT with (Es)Ketamine is an innovative approach potentially offering a faster antidepressant effect. However, evidence does not support a sustained long-term benefit. Further randomized studies with larger samples, standardized dosing protocols, and long-term outcome are required. Treatment choice should be individualized according to clinical profile, expected response, and tolerability.

**Disclosure of Interest:**

None Declared

